# (*S*)-Alanine–(*S*)-2-phen­oxy­propionic acid (1/1)

**DOI:** 10.1107/S1600536812020727

**Published:** 2012-05-16

**Authors:** Kiichi Amimoto, Yuma Nishioka

**Affiliations:** a Department of Science Education, Graduate School of Education, Hiroshima University, 1-1-1 Kagamiyama, Higashi-Hiroshima, Hiroshima, Japan

## Abstract

In the title co-crystal, C_3_H_7_NO_2_·C_9_H_10_O_3_, the (*S*)-alanine mol­ecule exists in the zwitterionic form stabilized by two pairs of N^+^—H⋯O^−^ hydrogen bonds and an electrostatic inter­action between the ammonium center and the carboxyl­ate anion, forming a sheet along the *ab* plane. The carboxyl group of the (*S*)-2-phen­oxy­propionic acid mol­ecule is connected to the top and bottom of the sheet *via* N^+^—H⋯O=C and O—H⋯O^−^ [*R*_2_^2^(7) graph set] hydrogen bonds, giving an (*S*,*S*)-homochiral layer, in which both methyl groups of (*S*)-alanine and the phenyl rings of (*S*)-2-phen­oxy­propionic acid are oriented in the same direction along the *b* axis.

## Related literature

For the use of a chiral resolution agents, see: Hasegawa *et al.* (1998 ▶). For the crystal structure of enanti­omeric and racemic 2-phen­oxy­propionic acid, see: Sørensen & Larsen (2003 ▶). For the crystal structure of (*S*)-alanine–(*R*)-2-phen­oxy­propionic acid, see: Takahashi & Fujii (2004 ▶).
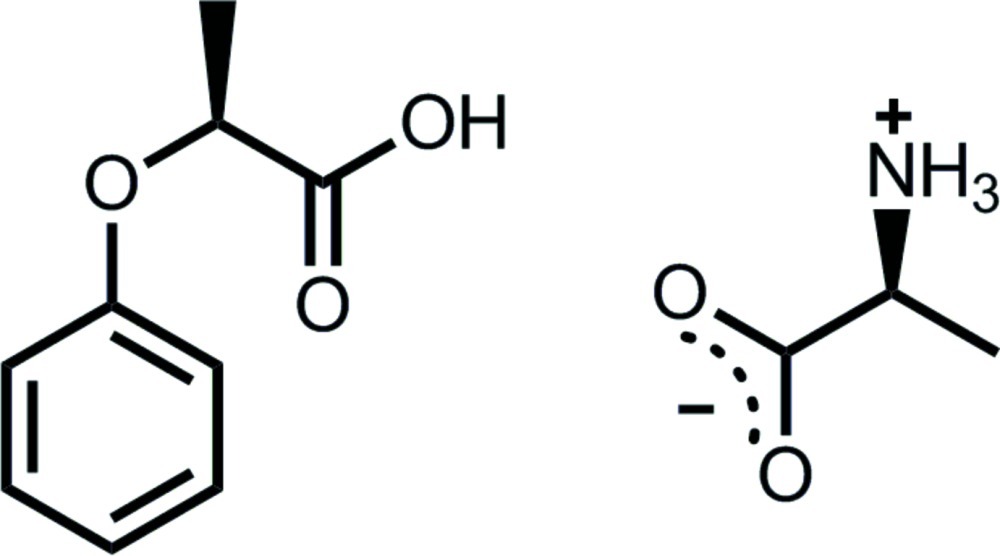


## Experimental

### 

#### Crystal data

C_3_H_7_NO_2_·C_9_H_10_O_3_
*M* *_r_* = 255.27
Monoclinic, 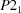

*a* = 5.227 (5) Å
*b* = 7.364 (5) Å
*c* = 17.493 (5) Å
β = 95.232 (5)°
*V* = 670.5 (8) Å^3^
*Z* = 2
Mo *K*α radiation
μ = 0.10 mm^−1^
*T* = 293 K
0.7 × 0.5 × 0.3 mm

#### Data collection

Bruker APEXII CCD area-detector diffractometer
3872 measured reflections
2742 independent reflections
2698 reflections with *I* > 2σ(*I*)
*R*_int_ = 0.021

#### Refinement

*R*[*F* ^2^ > 2σ(*F* ^2^)] = 0.029
*wR*(*F* ^2^) = 0.081
*S* = 1.06
2742 reflections
171 parameters
1 restraint
H atoms treated by a mixture of independent and constrained refinement
Δρ_max_ = 0.17 e Å^−3^
Δρ_min_ = −0.13 e Å^−3^
Absolute structure: Flack (1983 ▶), 930 Friedel pairs
Flack parameter: −0.4 (8)

### 

Data collection: *APEX2* (Bruker, 2009 ▶); cell refinement: *SAINT* (Bruker, 2009 ▶); data reduction: *SAINT*; program(s) used to solve structure: *SIR97* (Altomare *et al.*, 1999 ▶); program(s) used to refine structure: *SHELXL97* (Sheldrick, 2008 ▶); molecular graphics: *Mercury* (Macrae *et al.*, 2008 ▶) and *ORTEP-3* (Farrugia, 1997 ▶); software used to prepare material for publication: *WinGX* (Farrugia, 1999 ▶) and *publCIF* (Westrip, 2010 ▶).

## Supplementary Material

Crystal structure: contains datablock(s) I, global. DOI: 10.1107/S1600536812020727/ds2190sup1.cif

Structure factors: contains datablock(s) I. DOI: 10.1107/S1600536812020727/ds2190Isup2.hkl

Supplementary material file. DOI: 10.1107/S1600536812020727/ds2190Isup3.cml

Additional supplementary materials:  crystallographic information; 3D view; checkCIF report

## Figures and Tables

**Table 1:** Hydrogen-bond geometry (Å, °)

*D*—H⋯*A*	*D*—H	H⋯*A*	*D*⋯*A*	*D*—H⋯*A*
N1—H1*A*⋯O5^i^	0.89	2.05	2.916 (3)	165
N1—H1*B*⋯O5^ii^	0.89	1.94	2.822 (3)	170
N1—H1*C*⋯O2^i^	0.89	1.97	2.863 (3)	177
O1—H1*O*⋯O4^ii^	1.03 (2)	1.50 (2)	2.521 (3)	169 (2)

Symmetry codes: (i) 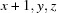; (ii) 
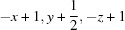
.

**Fig. 1.**
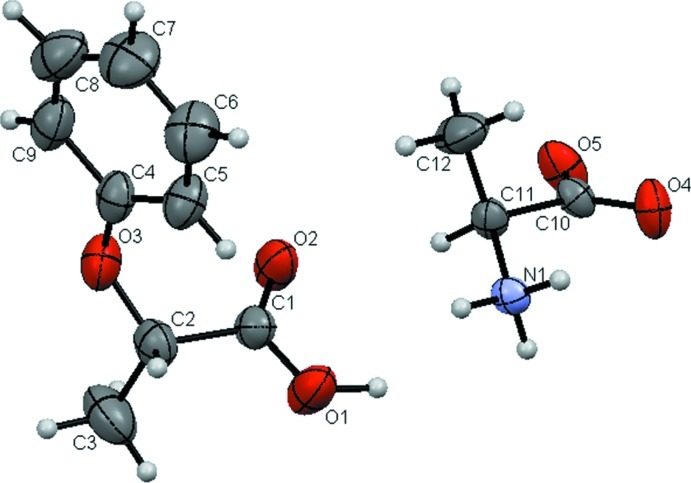
The moleculer structure of the title compound I, showing the atom-labelling scheme and displacement ellipsoids at the 50% probability level.

**Fig. 2.**
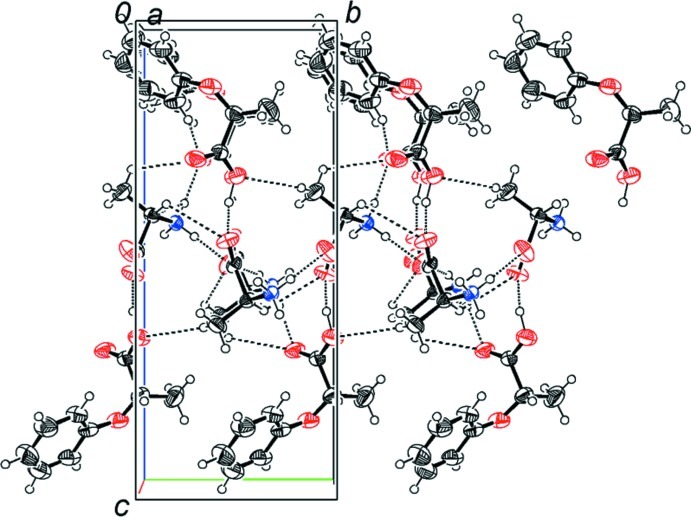
A view of the crystal packing of the title compound I, viewed down the c axis (anisotropic displacement ellipsoids drawn at 50% probability level). Hydrogen bonds are drawn as dashed lines.

## supplementary crystallographic information

### Comment

Chiral 2-phenoxypropionic acid (PPA) has been known as a good and accessible optical resolving agent for amines (Hasegawa *et al.*, 1998). The crystal structure of optical pure and racemic PPA has been reported (Sørensen & Larsen, 2003). And the crystal structure of co-crystal of (*R*)-PPA with (*S*)-alanine has been known (Takahashi & Fujii, 2004), but no results have ever reported on the details of the chiral discrimination between PPA and (*S*)-alanine. In this work, we present the crystal structure of the co-crystal of (*S*)-PPA with (*S*)-alanine (I) (Fig. 1). The co-crystal I crystallizes in the monoclinic system of space group *P*2_1_. (*S*)-Alanine assembles a chiral two-dimensional sheet along the *ab* plane, in which the ammonium cation is strongly held with the carboxylate anion by two hydrogen bonds and one electrostatic interaction. The N(1)^+^—H···O(5)^-^ hydrogen bonds are 2.822 (2) Å and 2.916 (2) Å. The interatomic distance between ammonium center N(1)^+^ and carboxylate O(4)^-^ is 2.960 (2) Å. The carboxyl C=O of PPA is connected to the ammonium N^+^—H of (*S*)-alanine, and the O—H of PPA to the carboxylate O^-^ of (*S*)-alanine. The N(1)^+^—H···O(2) and O(1)—H···O(4)^-^ distances are 2.863 (2) Å and 2.521 (2) Å, respectively. The phenyl ring of PPA is oriented in the same direction of the methyl group of (*S*)-alanine of chiral two-dimensional sheet, yielding a (*S*,*S*)-homochiral layer (Fig. 2). On the basis of this finding, the development of optical resolution of amino acid using PPA as an optical resolving agent is under investigation.

### Experimental

All reagents were commercially available from WAKO Co. and used without purification.(*S*)-2-Phenoxypropionic acid (1.66 g, 10 mmol) and (*S*)-alanine (0.89 g, 10 mmol) were dissolved in a water/ethanol solution (10 ml, 1:1 *v*/*v*). The solution was refluxed for 10 min, cooled to room temperature, and then kept in the refrigerator for three days. Colorless single crystals of I were obtained that were suitable for X-ray diffraction study.

### Refinement

All hydrogen atoms were found in a difference Fourier map. The hydrogen atom of carboxylic O(1)—H was refined isotropically. Other hydrogen atoms were refined as riding atoms with C_aromatic_—H = 0.93 Å, C_methyl_—H = 0.96 Å, and C_methine_—H = 0.98 Å, and with *U*_iso_(H) = 1.5*U*_eq_(C_methyl_) and *U*_iso_(H) = 1.2*U*_eq_(C_aromatic_, C_methine_), respectively.

### Crystal data

**Table:**

C_3_H_7_NO_2_·C_9_H_10_O_3_	*F*(000) = 272
*M**_r_* = 255.27	*D*_x_ = 1.264 Mg m^−^^3^
Monoclinic, *P*2_1_	Mo *K*α radiation, λ = 0.71069 Å
Hall symbol: P 2yb	Cell parameters from 3229 reflections
*a* = 5.227 (5) Å	θ = 2.3–28.5°
*b* = 7.364 (5) Å	µ = 0.10 mm^−^^1^
*c* = 17.493 (5) Å	*T* = 293 K
β = 95.232 (5)°	Plate, colourless
*V* = 670.5 (8) Å^3^	0.7 × 0.5 × 0.3 mm
*Z* = 2	

### Data collection

**Table:**

Bruker APEXII CCD area-detector diffractometer	2698 reflections with *I* > 2σ(*I*)
Radiation source: fine-focus sealed tube	*R*_int_ = 0.021
Graphite monochromator	θ_max_ = 28.5°, θ_min_ = 2.3°
Detector resolution: 8.333 pixels mm^-1^	*h* = −6→5
phi and ω scan	*k* = −9→9
3872 measured reflections	*l* = −21→23
2742 independent reflections	

### Refinement

**Table:**

Refinement on *F*^2^	Hydrogen site location: inferred from neighbouring sites
Least-squares matrix: full	H atoms treated by a mixture of independent and constrained refinement
*R*[*F*^2^ > 2σ(*F*^2^)] = 0.029	*w* = 1/[σ^2^(*F*_o_^2^) + (0.0452*P*)^2^ + 0.0566*P*] where *P* = (*F*_o_^2^ + 2*F*_c_^2^)/3
*wR*(*F*^2^) = 0.081	(Δ/σ)_max_ < 0.001
*S* = 1.06	Δρ_max_ = 0.17 e Å^−^^3^
2742 reflections	Δρ_min_ = −0.13 e Å^−^^3^
171 parameters	Extinction correction: *SHELXL97* (Sheldrick, 2008)
1 restraint	Extinction coefficient: 0.160 (9)
Primary atom site location: structure-invariant direct methods	Absolute structure: Flack (1983), 930 Friedel pairs
Secondary atom site location: difference Fourier map	Flack parameter: −0.4 (8)

### Special details

**Table:**

Geometry. All e.s.d.'s (except the e.s.d. in the dihedral angle between two l.s. planes) are estimated using the full covariance matrix. The cell e.s.d.'s are taken into account individually in the estimation of e.s.d.'s in distances, angles and torsion angles; correlations between e.s.d.'s in cell parameters are only used when they are defined by crystal symmetry. An approximate (isotropic) treatment of cell e.s.d.'s is used for estimating e.s.d.'s involving l.s. planes.
Refinement. Refinement of *F*^2^ against ALL reflections. The weighted *R*-factor *wR* and goodness of fit *S* are based on *F*^2^, conventional *R*-factors *R* are based on *F*, with *F* set to zero for negative *F*^2^. The threshold expression of *F*^2^ > σ(*F*^2^) is used only for calculating *R*-factors(gt) *etc*. and is not relevant to the choice of reflections for refinement. *R*-factors based on *F*^2^ are statistically about twice as large as those based on *F*, and *R*- factors based on ALL data will be even larger.

### Fractional atomic coordinates and isotropic or equivalent isotropic displacement parameters (Å^2^)

**Table:**

	*x*	*y*	*z*	*U*_iso_*/*U*_eq_	
C1	0.2142 (2)	0.91172 (17)	0.27450 (7)	0.0400 (3)	
C2	0.2315 (3)	0.9885 (2)	0.19397 (8)	0.0489 (3)	
H2	0.4105	1.0177	0.1867	0.059*	
C3	0.0685 (5)	1.1604 (3)	0.18625 (12)	0.0785 (6)	
H3A	0.0795	1.2125	0.1363	0.118*	
H3B	0.1306	1.2461	0.2249	0.118*	
H3C	−0.1071	1.1307	0.1926	0.118*	
C4	0.2830 (3)	0.7215 (2)	0.12044 (7)	0.0460 (3)	
C5	0.5113 (3)	0.6774 (3)	0.16181 (8)	0.0531 (3)	
H5	0.5723	0.7466	0.2041	0.064*	
C6	0.6486 (4)	0.5288 (3)	0.13974 (10)	0.0688 (5)	
H6	0.8015	0.4971	0.168	0.083*	
C7	0.5623 (4)	0.4276 (3)	0.07671 (12)	0.0776 (6)	
H7	0.6572	0.3286	0.0622	0.093*	
C8	0.3355 (4)	0.4726 (3)	0.03498 (10)	0.0708 (5)	
H8	0.2773	0.4045	−0.0079	0.085*	
C9	0.1955 (3)	0.6180 (2)	0.05671 (8)	0.0572 (4)	
H9	0.0412	0.6477	0.0288	0.069*	
C10	0.5537 (2)	0.48177 (15)	0.48456 (7)	0.0332 (2)	
C11	0.6485 (2)	0.55376 (16)	0.41011 (6)	0.0337 (2)	
H11	0.5119	0.6252	0.3823	0.04*	
C12	0.7222 (4)	0.3969 (2)	0.35972 (9)	0.0634 (4)	
H12A	0.7836	0.4441	0.3136	0.095*	
H12B	0.5746	0.3217	0.3468	0.095*	
H12C	0.855	0.3261	0.387	0.095*	
N1	0.87704 (17)	0.67096 (13)	0.42842 (5)	0.0322 (2)	
H1A	1.0014	0.6064	0.4537	0.048*	
H1B	0.835	0.7636	0.4574	0.048*	
H1C	0.9317	0.7131	0.3851	0.048*	
O1	0.3772 (2)	0.98974 (17)	0.32449 (6)	0.0594 (3)	
O2	0.0633 (2)	0.79618 (16)	0.28881 (5)	0.0556 (3)	
O3	0.1324 (2)	0.86666 (16)	0.13631 (5)	0.0543 (3)	
O4	0.72311 (17)	0.45179 (16)	0.53819 (5)	0.0478 (2)	
O5	0.32016 (16)	0.45273 (15)	0.48470 (6)	0.0505 (3)	
H1O	0.348 (4)	0.961 (4)	0.3808 (13)	0.090 (7)*	

### Atomic displacement parameters (Å^2^)

**Table:**

	*U*^11^	*U*^22^	*U*^33^	*U*^12^	*U*^13^	*U*^23^
C1	0.0455 (6)	0.0409 (6)	0.0347 (5)	−0.0023 (5)	0.0092 (5)	0.0001 (5)
C2	0.0571 (8)	0.0507 (7)	0.0405 (6)	−0.0004 (6)	0.0123 (5)	0.0086 (6)
C3	0.1025 (15)	0.0610 (10)	0.0741 (11)	0.0222 (10)	0.0188 (11)	0.0239 (9)
C4	0.0487 (7)	0.0597 (8)	0.0306 (5)	−0.0026 (6)	0.0090 (5)	0.0051 (5)
C5	0.0523 (8)	0.0688 (9)	0.0387 (6)	0.0046 (7)	0.0064 (5)	−0.0053 (7)
C6	0.0626 (10)	0.0849 (13)	0.0604 (9)	0.0170 (9)	0.0138 (8)	−0.0034 (9)
C7	0.0906 (14)	0.0761 (13)	0.0697 (11)	0.0092 (11)	0.0269 (10)	−0.0153 (10)
C8	0.0871 (12)	0.0749 (11)	0.0527 (8)	−0.0212 (10)	0.0184 (8)	−0.0165 (8)
C9	0.0613 (9)	0.0745 (10)	0.0355 (6)	−0.0130 (8)	0.0029 (6)	0.0007 (6)
C10	0.0292 (5)	0.0326 (5)	0.0387 (5)	0.0014 (4)	0.0078 (4)	0.0082 (4)
C11	0.0309 (5)	0.0378 (5)	0.0321 (5)	−0.0065 (4)	0.0009 (4)	0.0051 (4)
C12	0.0933 (12)	0.0534 (8)	0.0457 (7)	−0.0229 (8)	0.0179 (8)	−0.0170 (6)
N1	0.0313 (4)	0.0328 (4)	0.0335 (4)	−0.0041 (4)	0.0076 (3)	0.0015 (4)
O1	0.0697 (7)	0.0685 (7)	0.0406 (5)	−0.0264 (6)	0.0089 (5)	−0.0073 (5)
O2	0.0657 (7)	0.0647 (6)	0.0372 (5)	−0.0238 (5)	0.0086 (4)	0.0042 (4)
O3	0.0563 (6)	0.0709 (7)	0.0348 (4)	0.0084 (5)	−0.0010 (4)	0.0028 (4)
O4	0.0364 (4)	0.0718 (7)	0.0358 (4)	0.0054 (4)	0.0060 (3)	0.0173 (5)
O5	0.0288 (4)	0.0571 (5)	0.0663 (6)	−0.0030 (4)	0.0082 (4)	0.0240 (5)

### Geometric parameters (Å, º)

**Table:**

C1—O2	1.2019 (17)	C7—H7	0.93
C1—O1	1.2980 (18)	C8—C9	1.370 (3)
C1—C2	1.5281 (17)	C8—H8	0.93
C2—O3	1.4124 (19)	C9—H9	0.93
C2—C3	1.525 (3)	C10—O5	1.2398 (18)
C2—H2	0.98	C10—O4	1.2494 (16)
C3—H3A	0.96	C10—C11	1.5298 (15)
C3—H3B	0.96	C11—N1	1.4848 (17)
C3—H3C	0.96	C11—C12	1.524 (2)
C4—O3	1.3708 (19)	C11—H11	0.98
C4—C5	1.377 (2)	C12—H12A	0.96
C4—C9	1.393 (2)	C12—H12B	0.96
C5—C6	1.383 (3)	C12—H12C	0.96
C5—H5	0.93	N1—H1A	0.89
C6—C7	1.373 (3)	N1—H1B	0.89
C6—H6	0.93	N1—H1C	0.89
C7—C8	1.375 (3)	O1—H1O	1.03 (2)
			
O2—C1—O1	125.16 (12)	C9—C8—H8	120.1
O2—C1—C2	123.29 (12)	C7—C8—H8	120.1
O1—C1—C2	111.53 (12)	C8—C9—C4	120.31 (18)
O3—C2—C3	107.36 (15)	C8—C9—H9	119.8
O3—C2—C1	112.01 (12)	C4—C9—H9	119.8
C3—C2—C1	108.02 (12)	O5—C10—O4	126.77 (11)
O3—C2—H2	109.8	O5—C10—C11	117.18 (11)
C3—C2—H2	109.8	O4—C10—C11	115.99 (10)
C1—C2—H2	109.8	N1—C11—C12	108.94 (11)
C2—C3—H3A	109.5	N1—C11—C10	109.53 (9)
C2—C3—H3B	109.5	C12—C11—C10	110.39 (11)
H3A—C3—H3B	109.5	N1—C11—H11	109.3
C2—C3—H3C	109.5	C12—C11—H11	109.3
H3A—C3—H3C	109.5	C10—C11—H11	109.3
H3B—C3—H3C	109.5	C11—C12—H12A	109.5
O3—C4—C5	124.33 (13)	C11—C12—H12B	109.5
O3—C4—C9	115.83 (14)	H12A—C12—H12B	109.5
C5—C4—C9	119.82 (15)	C11—C12—H12C	109.5
C4—C5—C6	119.16 (15)	H12A—C12—H12C	109.5
C4—C5—H5	120.4	H12B—C12—H12C	109.5
C6—C5—H5	120.4	C11—N1—H1A	109.5
C7—C6—C5	120.85 (19)	C11—N1—H1B	109.5
C7—C6—H6	119.6	H1A—N1—H1B	109.5
C5—C6—H6	119.6	C11—N1—H1C	109.5
C6—C7—C8	120.01 (19)	H1A—N1—H1C	109.5
C6—C7—H7	120	H1B—N1—H1C	109.5
C8—C7—H7	120	C1—O1—H1O	114.0 (14)
C9—C8—C7	119.85 (17)	C4—O3—C2	117.36 (12)

### Hydrogen-bond geometry (Å, º)

**Table:**

*D*—H···*A*	*D*—H	H···*A*	*D*···*A*	*D*—H···*A*
N1—H1*A*···O5^i^	0.89	2.05	2.916 (3)	165
N1—H1*B*···O5^ii^	0.89	1.94	2.822 (3)	170
N1—H1*C*···O2^i^	0.89	1.97	2.863 (3)	177
O1—H1*O*···O4^ii^	1.03 (2)	1.50 (2)	2.521 (3)	169 (2)

Symmetry codes: (i) *x*+1, *y*, *z*; (ii) −*x*+1, *y*+1/2, −*z*+1.
